# Targeting cancer initiating cells by promoting cell differentiation and restoring chemosensitivity via dual inactivation of STAT3 and Src activity using an active component of *Antrodia cinnamomea* mycelia

**DOI:** 10.18632/oncotarget.12194

**Published:** 2016-09-22

**Authors:** Ching-Wen Chang, Yu-Syuan Chen, Chien-Chih Chen, Ik-On Chan, Chin-Chu Chen, Sen-Je Sheu, Ting-wei Lin, Shiu-Huey Chou, Chung-Ji Liu, Te-Chang Lee, Jeng-Fan Lo

**Affiliations:** ^1^ Institute of Oral Biology, National Yang-Ming University, Taipei, Taiwan; ^2^ Department of Biotechnology, Hungkuang University, Taichung, Taiwan; ^3^ Grape King Inc., Taoyuan County, Taiwan; ^4^ Department of Life Science, Fu-Jen University, Taipei, Taiwan; ^5^ Department of Oral and Maxillofacial Surgery, Mackay Memorial Hospital, Taipei, Taiwan; ^6^ Institute of Biomedical Sciences, Academia Sinica, Taipei, Taiwan; ^7^ Graduate Institute of Chinese Medical Science and Institute of Medical Science, China Medical University, Taichung, Taiwan; ^8^ Genome Research Center, National Yang-Ming University, Taipei, Taiwan; ^9^ Department of Dentistry, Taipei Veterans General Hospital, Taipei, Taiwan

**Keywords:** ergone, cancer initiating cells, STAT3, Src, differentiation

## Abstract

Cancer initiating cells (CICs) represent a subpopulation of cancer cells, which are responsible for tumor growth and resistance to chemotherapy. Herein, we first used a cell-based aldehyde dehydrogenase (ALDH) activity assay to identify that YMGKI-2 (also named as Ergone), an active component purified from *Antrodia cinnamomea* Mycelia extract (ACME), effectively abrogated the ALDH activity and abolished the CICs in head and neck squamous cell carcinoma cells (HNSCCs). Consequently, YMGKI-2 treatment suppressed self-renewal ability and expression of stemness signature genes (Oct-4 and Nanog) of sphere cells with enriched CICs. Moreover, YMGKI-2 treated sphere cells displayed reduction of CICs properties and promotion of cell differentiation, but not significant cytotoxicity. YMGKI-2 treatment also attenuated the tumorigenicity of HNSCC cells *in vivo*. Mechanistically, treatment of YMGKI-2 resulted in inactivation of STAT3 and Src. Lastly, combinatorial treatments with YMGKI-2 and standard chemotherapeutic drugs (cisplatin or Fluorouracil) restored the chemosensivity on sphere cells and cisplatin-resistant HNSCC cells. Together, we demonstrate that YMGKI-2 treatment effectively induces differentiation and reduces tumorigenicity of CICs. Further, combined treatment of YMGKI-2 and conventional chemotherapy can overcome chemoresistance. These results suggest that YMGKI-2 treatment may be used to improve future clinical responses in head and neck cancer treatment through targeting CICs.

## INTRODUCTION

Head and neck squamous cell carcinoma (HNSCC) represents the sixth most common cancer with an estimated 600,000 new cases annually worldwide [1]. The treatment outcome for patients with HNSCC remains poor with five-year survival rate of above 50% [2]. HNSCC-related death is due to lymph node invasion, metastasis and acquired resistance to conventional therapy [2]. Despite improvements in the diagnosis and treatment of HNSCC patients, the overall long-term survival rate remains dismal [2]. It is imperative to establish the novel therapeutic regime focusing on unconventional targets to cope with chemoresistance and prolong the life of HNSCC patients.

Accumulating data demonstrates that cancer-initiating cells (CICs), a subset of cancer cells with stem cell properties, are involved in tumor progression, metastasis and resistance to conventional therapies [3–7]. Previously, we successfully identify the existence and enrich the subpopulation of head and neck cancer-initiating cells (HN-CICs) from HNSCC by sphere formation. We also demonstrate that sphere cells display enhanced CICs properties and tumorigenic potentials, etc. [8]. In addition, we discover that HN-CICs not only possess high aldehyde dehydrogenase (ALDH) activity but also, mainly, overlap with the same subpopulation of cells expressing CICs surface markers such as ^mem^Grp78, CD133 and Glut3 [9]. ALDH, a group of intracellular enzymes protects cells by catalyzing the oxidation of toxic agents [10, 11]. Others report that increased ALDH activity in CICs mediates the chemoresistance [12]. Therefore, the development of chemical compounds that effectively inhibit the ALDH activity may provide significant therapeutic benefits to HNSCC patients through targeting the CICs.

*Antrodia cinnamomea*, a rare medical mushroom of the family *Polyporaceae*, mainly, grows in Taiwan [13]. For Taiwanese medical herb, *Antrodia cinnamomea* has been widely applied for diarrhea, intoxication, hepatoprotection, itchy skin [14], and cancer prevention [15]. The biological activities of the crude extracts or purified components from fruiting bodies or submerged cultured mycelia of *Antrodia cinnamomea* have been identified [15]. Empirically, these active components in the fruiting body of *Antrodia cinnamomea* showed antitumor activities for several types of human cancer [16–19]. However, the inhibitory effect of *Antrodia cinnamomea* on the CICs remains unclear. Herein, we are interested in screening for the active components from *Antrodia cinnamomea* on targeting CICs and in clarifying the possible biological mechanisms to mediate the antitumor effects. Firstly, we used the cell-based ALDH activity assay to screen for the active components from *Antrodia cinnamomea* Mycelia extracts (ACMEs) on targeting cancer initiating cells. In fact, we have previously found that YMGKI-1, one of the active components from *Antrodia cinnamomea*, can inhibit CICs properties through inducing exaggerated autophagic cell death [20]. Indeed, several purified compounds from ACME may possess the biological activity for diminished CICs by which regulation of different signaling pathways to achieve its anticancer activity.

YMGKI-2 (also named ergone; ergosta-4,6,8(14),22-tetraen-3-one) is a well-known bioactive steroid and has been isolated from *Antrodia cinnamomea* [21]. YMGKI-2, a metabolite of ergosterol, ergosterol peroxide and ergosta-6,22-diene-3β,5α,8α-triol, is catalyzed by a set of multiple pathways [27]. Recent studies report that YMGKI-2 displays cytotoxic activity on cancer cells [22], diuretic activity [23], inhibition of nitric oxide production [24] and immunomodulating activity [25, 26]. For the cancer pharmacology, Zhao et al have found YMGKI-2 displays more cytotoxic effects on cancer cells than normal cells [27]. However, the anti-cancerous role of YMGKI-2 in CICs has not been well characterized.

In the present study, we showed that YMGKI-2, one of the active components from ACME, effectively inhibited the ALDH activity of CICs. YMGKI-2 reduced self-renewal ability and promotes differentiation but not caused significant cytotoxicity of CICs. Further, combined treatment of YMGKI-2 with chemotherapeutic agents displayed synergistic cytotoxicity on killing both sphere cells and chemoresistant HNSCC cells. Thus, YMGKI-2 may be a novel adjuvant drug for improvement of head and neck cancer treatment in the future.

## RESULTS

### Diminished cancer-initiating cells properties but without cytotoxic effect of YMGKI-2 (Ergone) treated HNSCC cells or sphere cells

**Figure 1:**
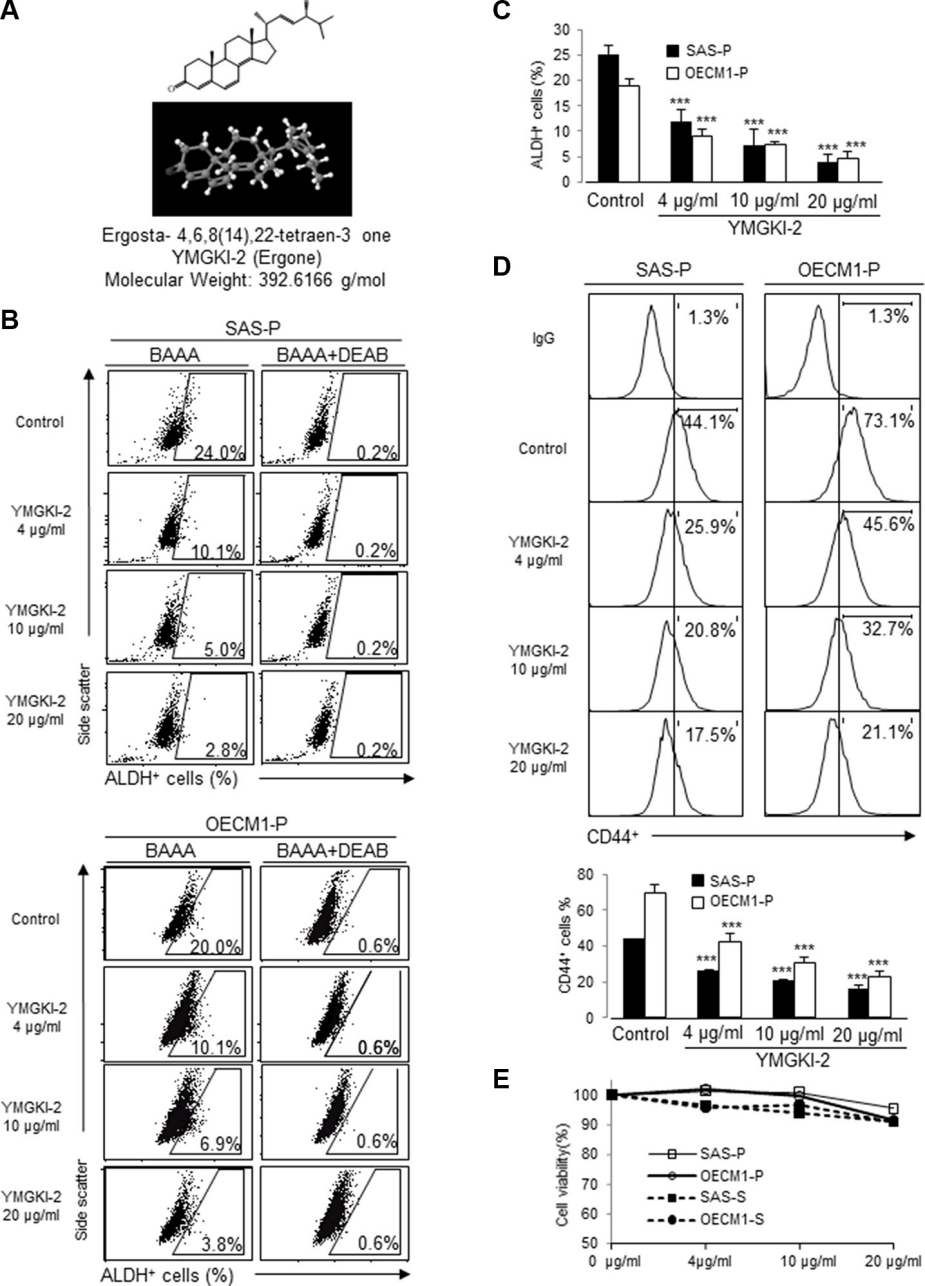
Reduced CICs subpopulation but not cytotoxic effect of YMGKI-2 treated HNSCC cells or sphere cells (**A**) Chemical structure of YMGKI-2 (Ergone) isolated from the mycelium of *Antrodia cinnamomea*. (**B**) HNSCC cells (SAS-P and OECM1-P) were treated with YMGKI-2 for 24 hrs, afterward; the intracellular ALDH activity was examined by ALDEFLUOR™ flow cytometry-based assay. BODIPY-aminoacetaldehyde (BAAA): BAAA is a fluorescent substrate for ALDH. DEAB, a specific inhibitor of ALDH1 enzyme, was used as negative control. (**C**) The bar graph shows quantification of ALDH-positive cells from panel (B). (**D**) Expression profile of CD44-positive cells of YMGKI-2 treated HNSCC cells was analyzed by flow cytometry. The bar graph shows quantification of CD44-positive cells. (**E**) Parental cells (SAS-P or OECM1-P) or sphere cells (SAS-S or OECM1-S) were treated with 0, 4, 10 or 20 μg/ml of YMGKI-2 for 24 hr, afterward, stained with propidium iodide (PI) and then examined by flow cytometry. The PI-negative cells were recorded as viable cells. The data are means ± SD of three independent experiments (*p < 0.05).

To examine the effect of *Antrodia cinnamomea* mycelia extract (ACME) on targeting cancer-initiating cells, we used Aldehyde dehydrogenase (ALDH) activity assay to screen for the active components from ACME that can inhibit ALDH activity of HNSCC cells. Among the tested compounds, YMGKI-2 (Ergosta-4, 6, 8(14), 22-tetraen-3-one; Ergone) (Figure 1A)) treatment significantly reduced the ALDH enzymatic activity of HNSCC cell lines (SAS and OECM1) in a dose-dependent manner (Figure 1B and 1C). Our previous data demonstrate that membrane-anchoring GRP78 (^mem^GRP78) could be used as a surface marker for enrichment of HN-CICs [28]. To further verify whether the effect of YMGKI-2 treatment in disrupting HN-CIC, we determined the expression of ^mem^GRP78 in YMGKI-2 treated HNSCC cells. As expected, treatment of YMGKI-2 reduced the percentage of ^mem^GRP78 positive cells in HNSCC cells (Supplementary Figure S1A). Furthermore, the expression profile of CD44, an identified cell surface marker of CICs [3, 29], was also reduced after YMGKI-2 treatment in HNSCC cells (Figure 1D). To further determine whether treatment of YMGKI-2 inhibited the stemness properties through induction of cell death, parental (OECM1-P and SAS-P) and sphere cells with enriched HN-CICs (OECM1-S and SAS-S) were treated with YMGKI-2 and subjected to FACS analysis after propidium iodide (PI) staining. Interestingly, we observed that treatment of YMGKI-2 only caused slight cell death under high concentration conditions (Figure 1E). In addition, YMGKI-2 treatment did not cause significant cytotoxicity to normal human oral keratinocytes (NHOKs) (Supplementary Figure S1B). Hence, these findings suggest that YMGKI-2 treatment may effectively and specifically reduce CICs subpopulation but not cause significant cytotoxicity in HNSCCs, sphere cells and NHOKs.

### Reduced self-renewal ability and enhanced differentiation in YMGKI-2 treated sphere cells

**Figure 2:**
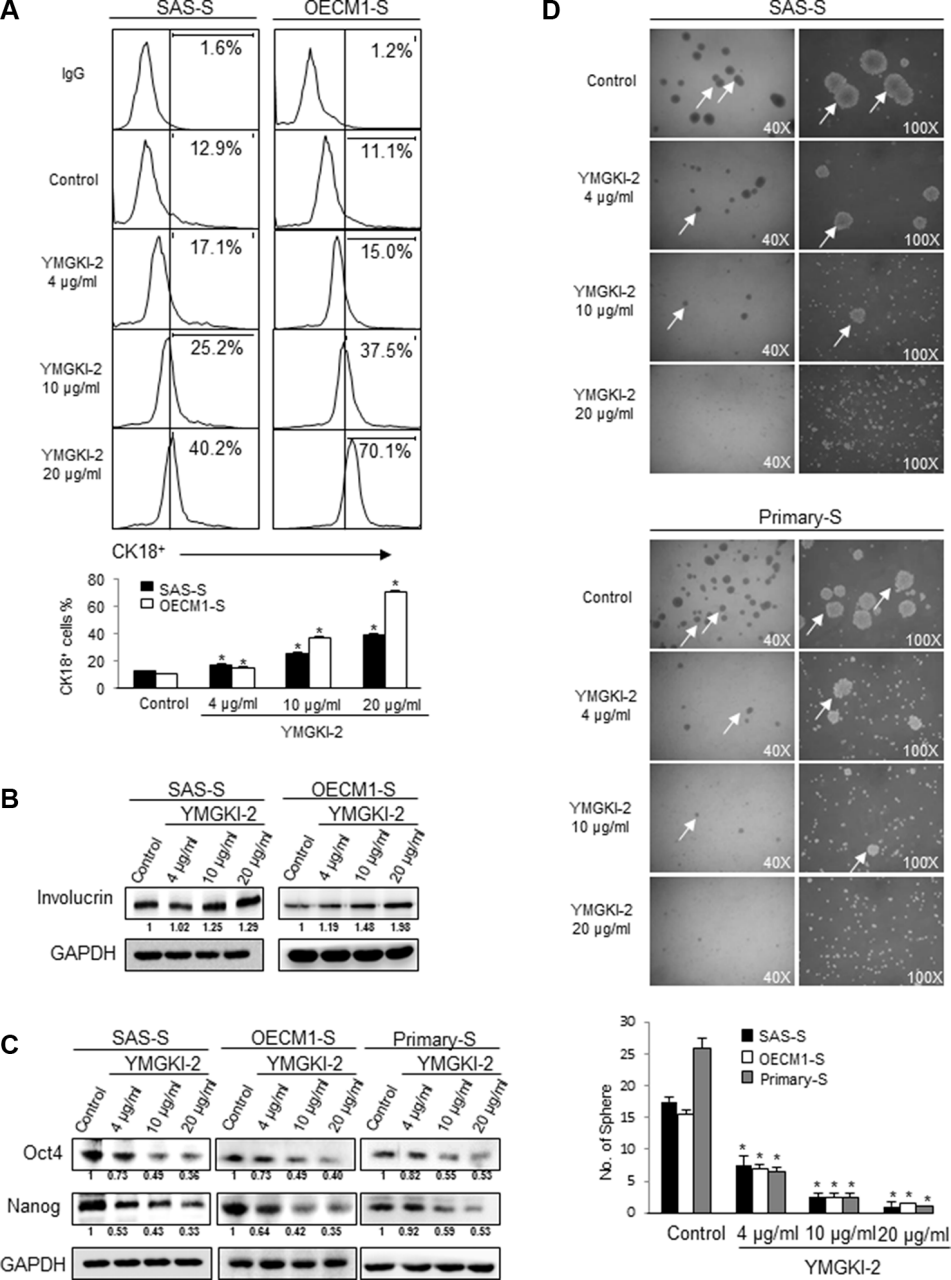
Diminished stemness properties and enhanced differentiation of sphere cells with YMGKI-2 treatment (**A**) Sphere cells (SAS-S and OECM1-S) were treated with YMGKI-2 at different concentration for 24 hrs, and then stained with anti-CK18 antibodies, secondary antibody conjugated with Cy5 fluorescence dye and detected by flow cytometry. The bar graph shows quantification of CK18-positive cells. (**B**) Immunoblots showing the expression of Involucrin and GAPDH in sphere cells with or without indicated treatments. (**C**) Crude cell extract proteins of YMGKI-2-treated sphere cells (SAS-S, OECM1-S and Primary-S) were collected and analyzed by immunoblotting against anti-Oct-4, anti-Nanog or anti-GAPDH antibodies as indicated. The immunoactive signal of GAPDH protein of different crude cell extracts was referred as loading control. (**D**) Sphere cells (SAS-S, OECM1-S and Primary-S) were treated with YMGKI-2 for 24 hrs, and the sphere formation ability of YMGKI-2 treated cells was examined. White arrows indicate the sphere body. The bar graph shows quantification of sphere number. Data are means ± SD of triplicate samples from three experiments (*P < 0.05).

Because the inhibitory effect of CIC properties by YMGKI-2 treatment was observed but not due to cell death in sphere cells (Figure 1E), we speculated that YMGKI-2 treatment might promote cell differentiation. As expected, sphere cells treated with YMGKI-2 displayed elevated expression of epithelial differentiation markers (CK18 (*P* < 0.05) [30] and Involucrin [28, 31]) (Figure 2A and 2B). Expression of stemness genes and sphere formation ability are the indexes for identifying CICs, and based on these properties to evaluate the self-renewal ability and undifferentiated status of CICs [3, 28]. Accordingly, we showed that protein level of stemness signature genes (Oct-4 and Nanog) was diminished in YMGKI-2 treated sphere cells including SAS-S, OECM1-S and Primary-S (established from the primary cells derived from HNSCC tumor tissue (see Materials and Methods)) by immunoblot analyses (Figure 2C). Additionally, the sphere formation ability of YMGKI-2 treated sphere cells was significantly abrogated in a dose-dependent manner (Figure 2D) (*P* < 0.05). Together, YMGKI-2 treatment not only promoted cell differentiation but also decreased self-renewal ability in sphere cells.

### Reduction of *in vitro* malignancy of sphere cells treated with YMGKI-2

**Figure 3:**
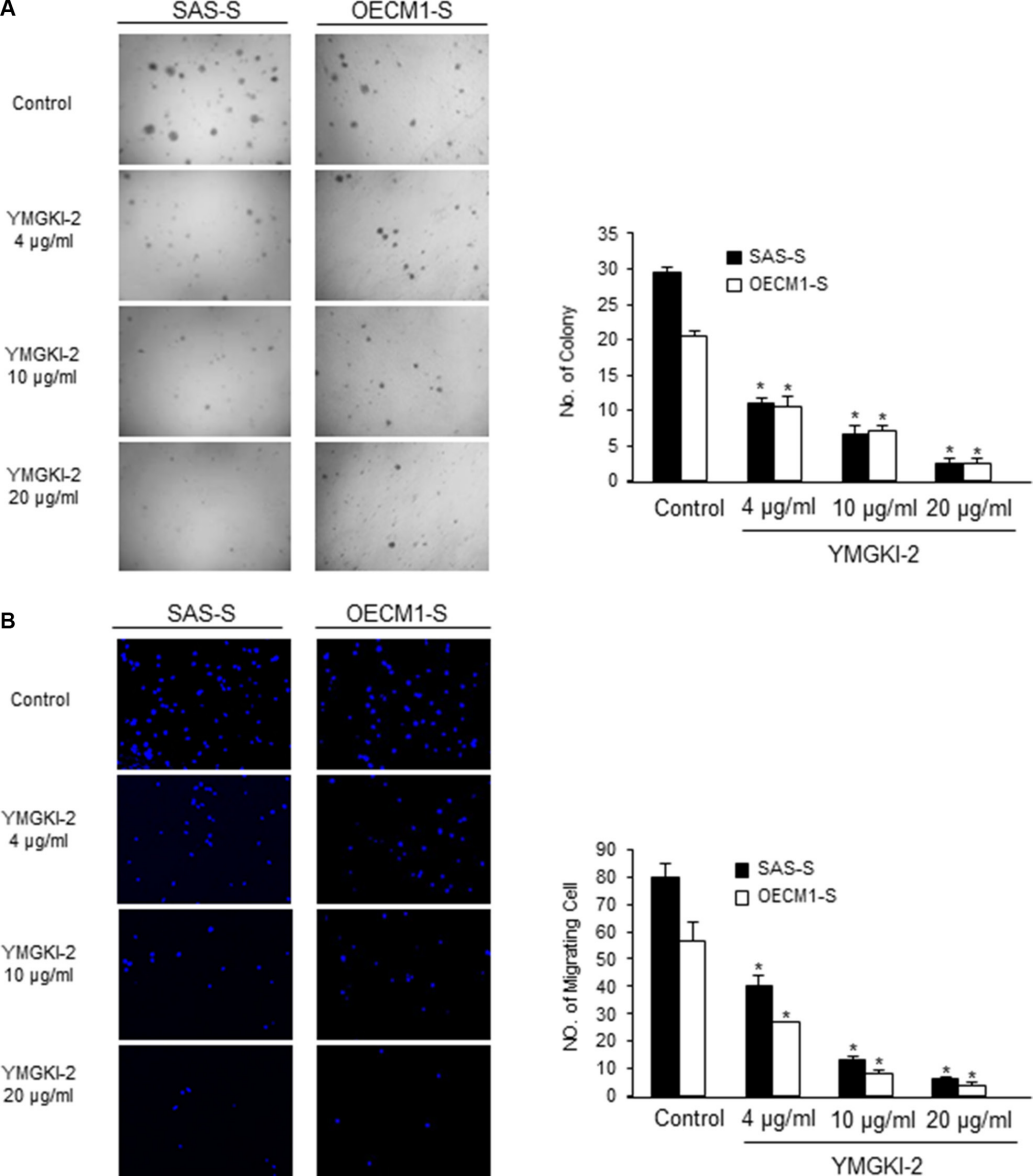
Reduced *in vitro* malignancy of sphere cells treated with YMGKI-2 (**A**) Sphere cells (SAS-S, OECM-1-S and Primary-S) treated with different concentration of YMGKI-2 for 24 hrs, then, were plated onto soft agar and grew for 12 day. The colony formation ability of the YMGKI-2 treated cells was examined. Data are means ± SD of triplicate samples from three experiments (*P* < 0.05). (**B**) Sphere cells (SAS-S, OECM1-S and Primary-S) treated with different concentration of YMGKI-2 for 24 hrs, afterwards, were plated onto Transwell. The migratory ability of YMGKI-2 treated cells was analyzed as described in Materials and Methods.

To determine whether YMGKI-2 treatment would affect the malignancy of enriched HN-CICs *in vitro*, we analyzed the anchorage independent growth potential and migration ability of YMGKI-2 treated sphere cells. As shown in Figure 3A and 3B, the anchorage independent growth potential and migration ability of YMGKI-2 treated sphere cells were significantly reduced. In summary, our results suggested that YMGKI-2 treatment reduced the *in vitro* malignancy of enriched HN-CICs.

### Attenuation on xenograft tumor growth of YMGKI-2 treated HNSCC *in vivo*

**Figure 4:**
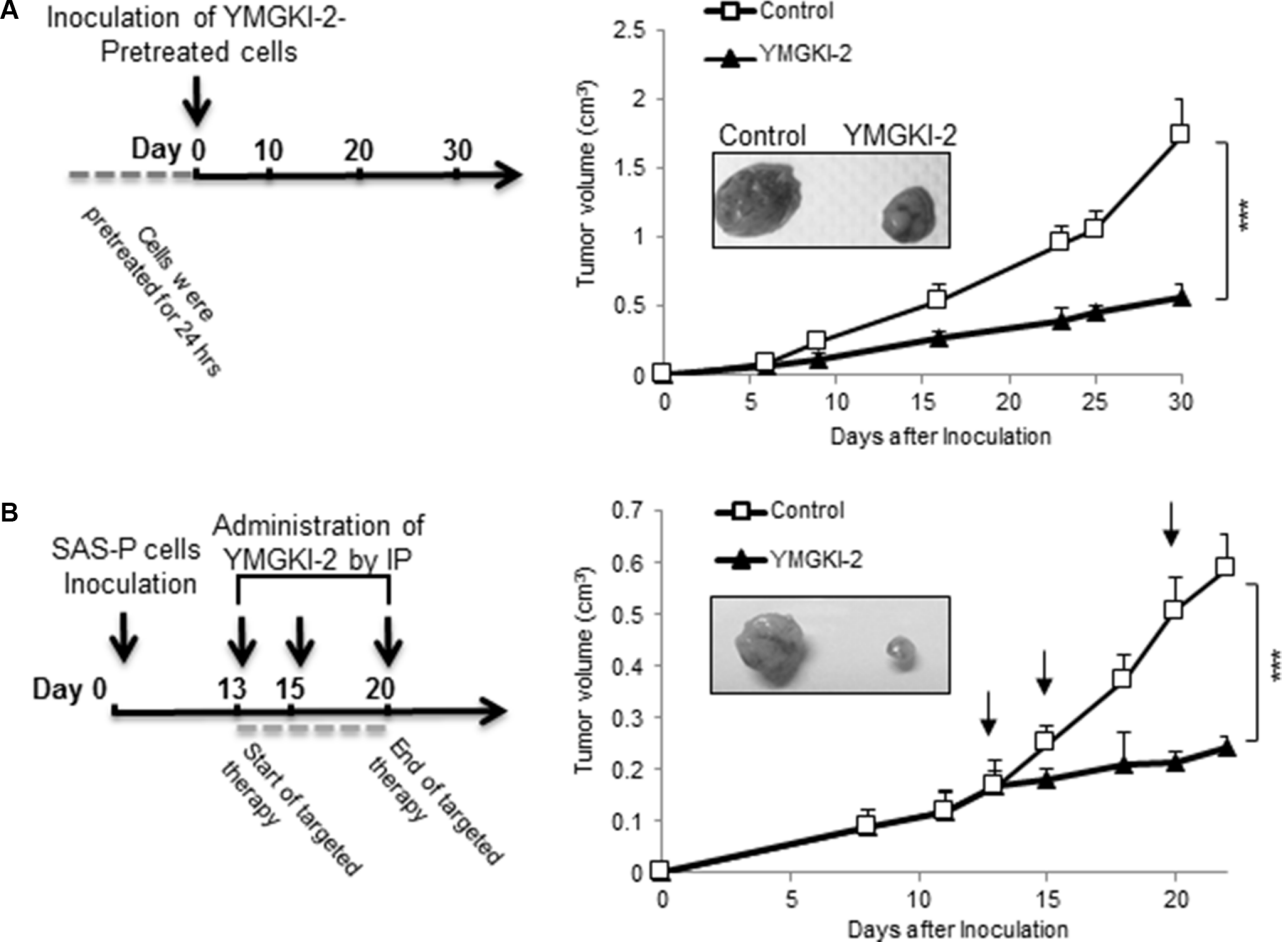
Attenuation on xenograft tumor growth of YMGKI-2 treated HNSCC *in vivo* (**A**) SAS cells pretreated with 10 μg/ml of YMGKI-2 for 24 hours were inoculated subcutaneously into nude mice. The curve of tumor growth and the representative image of dissected tumors 30 days afterward inoculation were recorded. (**B**) SAS cells (1 × 10^6^ cells) were subcutaneously inoculated into nude mice. When tumors became palpable, 20 mg/kg of YMGKI-2 was injected intraperitoneally on days 13, 15 and 20. Consequently, the tumor growth curves were recorded. Error bars correspond to SD. (*n* = 3; *P < 0.05).

To determine whether YMGKI-2 treatment attenuated the tumor-initiating ability of HNSCC cells *in vivo*, SAS cells (a tumorigenic cell line) with YMGKI-2 pretreatment were inoculated subcutaneously into nude mice (Figure 4A). As shown in Figure 4A, tumor development and growth from the SAS cells with YMGKI-2 pretreatment were significantly suppressed (***P < 0.005). Next, to test whether YMGKI-2 could function as an effective therapeutic reagent for HNSCC, we examined the effect of YMGKI-2 post-treatment on xenograft model of HNSCC. Experimentally, SAS cells were subcutaneously implanted into the back of nude mice and followed by the tumors formation. At day 13 after the cell inoculation, the tumor-bearing mice were intraperitoneally administered with ETOH (as control) or YMGKI-2, respectively. Effectively, tumor volume in all recipients that were administered with YMGKI-2 was reduced as compared with control group (Figure 4B) (*P* < 0.005). These data support that YMGKI-2 treatment attenuated tumor growth ability of HNSCC *in vivo*.

### Restored synergistic chemosensitivity of enriched HN-CICs by YMGKI-2 treatment

**Figure 5:**
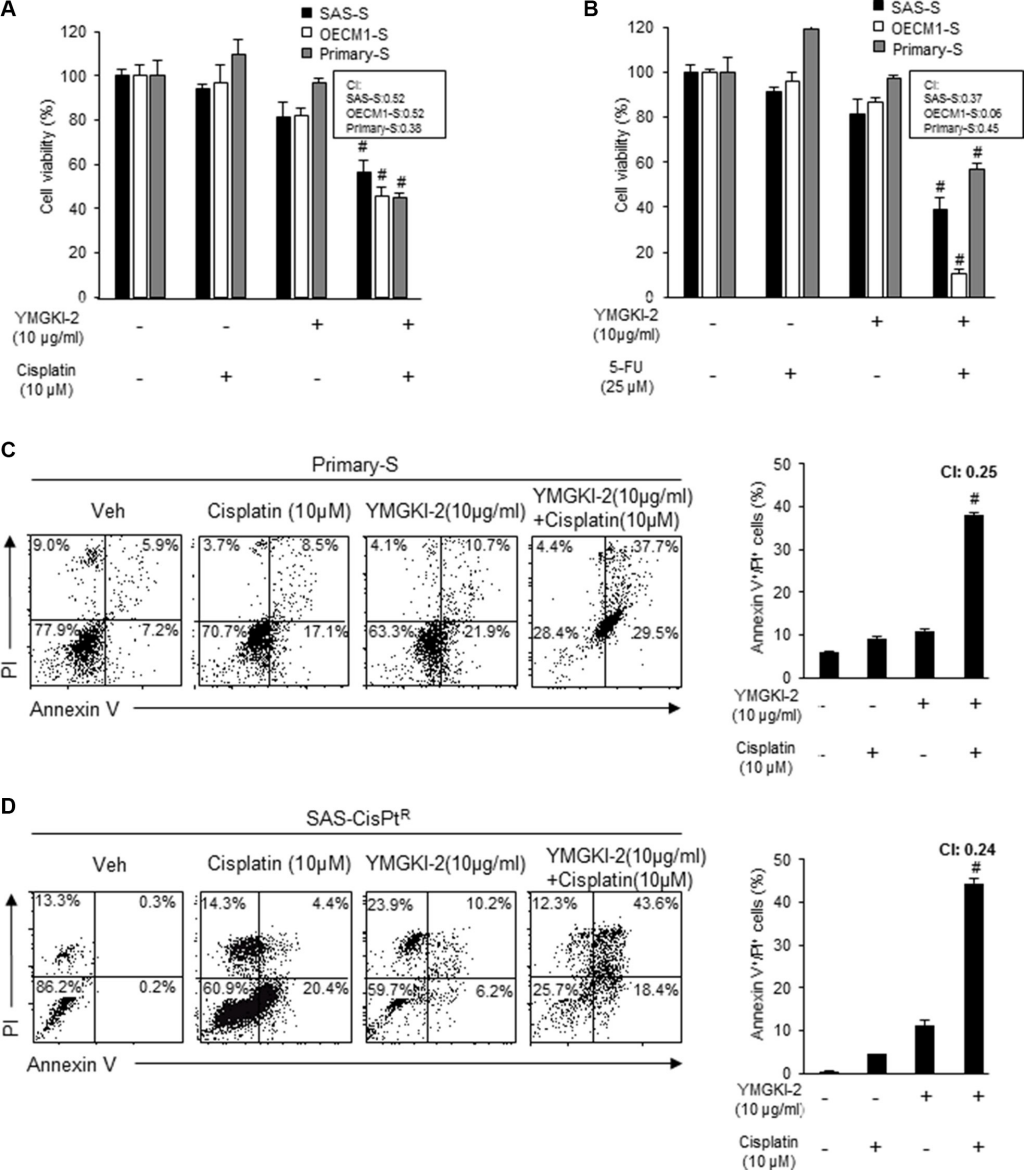
Restored chemosensitivity in YMGKI-2 treated sphere cells and cisplatin resistant cells Sphere cells (SAS-S, OECM1-S and Primary-S) were either singly treated with 10 μg/ml of YMGKI-2 or co-treated with (**A**) cisplatin (10 μM) or (**B)** 5-FU (25 μM). The cell viability was determined by MTT assay. The (**C**) sphere cells (Primary-S) or (**D**) SAS-cisplatin resistance cells (SAS-CisPt^R^) were either singly treated or co-treated with YMGKI-2 (10 μg/ml) and cisplatin (10 μM). The drug treated cells were co-stained with Annexin V and PI, then, the expression profile of the co-staining was collected (*left panels*). The percentage of cells positively stained with Annexin V and PI (Annexin V^+^/PI^+^) was plotted to indicate the dying cells (*right panels*). (^#^CI < 0; Combination index (CI)). Data are means ± SD of triplicate samples from three experiments (*P < 0.05).

Enriched HN-CICs have enhanced resistance to chemotherapeutic treatment including cisplatin and Fluorouracil (5-FU), which are used in patients with head and neck cancer [9, 32, 33]. Our current findings indicated that YMGKI-2 treatment diminished CICs properties. Therefore, it is reasonable to speculate that YMGKI-2 could act as an attenuator to interfere the chemoresistance of HN-CICs. To investigate whether YMGKI-2 treatment attenuated chemoresistance of CICs, we performed the single or combined treatment with YMGKI-2 plus chemotherapeutic agents (cisplatin or 5-FU) onto sphere cells (SAS-S, OECM1-S and Primary-S). The MTT assay indicated that sphere cells became highly sensitive to cisplatin or 5-FU in combined treatment with YMGKI-2 (Figure 5A and 5B). To further determine whether the reduction of cell viability in sphere cells with combined treatment of YMGKI-2 plus chemotherapeutic agents was due to induced apoptosis, we determined the status of cells undergoing cell death using Annexin V plus PI double staining. As shown in Figure 5C, the sphere cells (Primary-S) with the combined treatment of YMGKI-2 and chemotherapeutic agents displayed significant increase of apoptotic cells (Annexin V^+^/PI^+^) (Figure 5C). Further, up-regulation of CICs properties is significantly enhanced in cisplatin-resistant patients with head and neck cancer [34]. We have also shown that SAS-derived cisplatin resistant cells (SAS-Cis-Pt^R^) possessed increased CICs properties [9]. To study whether YMGKI-2 treatment was able to cope with the chemoresistance in HNSCC cells, we wanted to determine the cytotoxicity caused by co-treatment of YMGKI-2 and cisplatin to cisplatin resistant HNSCC cells *in vitro*. As shown in Figure 5D, the chemosensitivity of SAS-Cis-Pt^R^ cells to cisplatin, analyzed by Annexin V/PI double staining, was restored in combined treatment with YMGKI-2. Taken together, YMGKI-2 co-treatment exhibits a synergistic therapeutic effect in restoring the chemosensitivity by disrupting the CICs properties of head and neck cancer cells.

### Inhibition of Src and STAT3 activity in CICs by YMGKI-2 treatment

**Figure 6:**
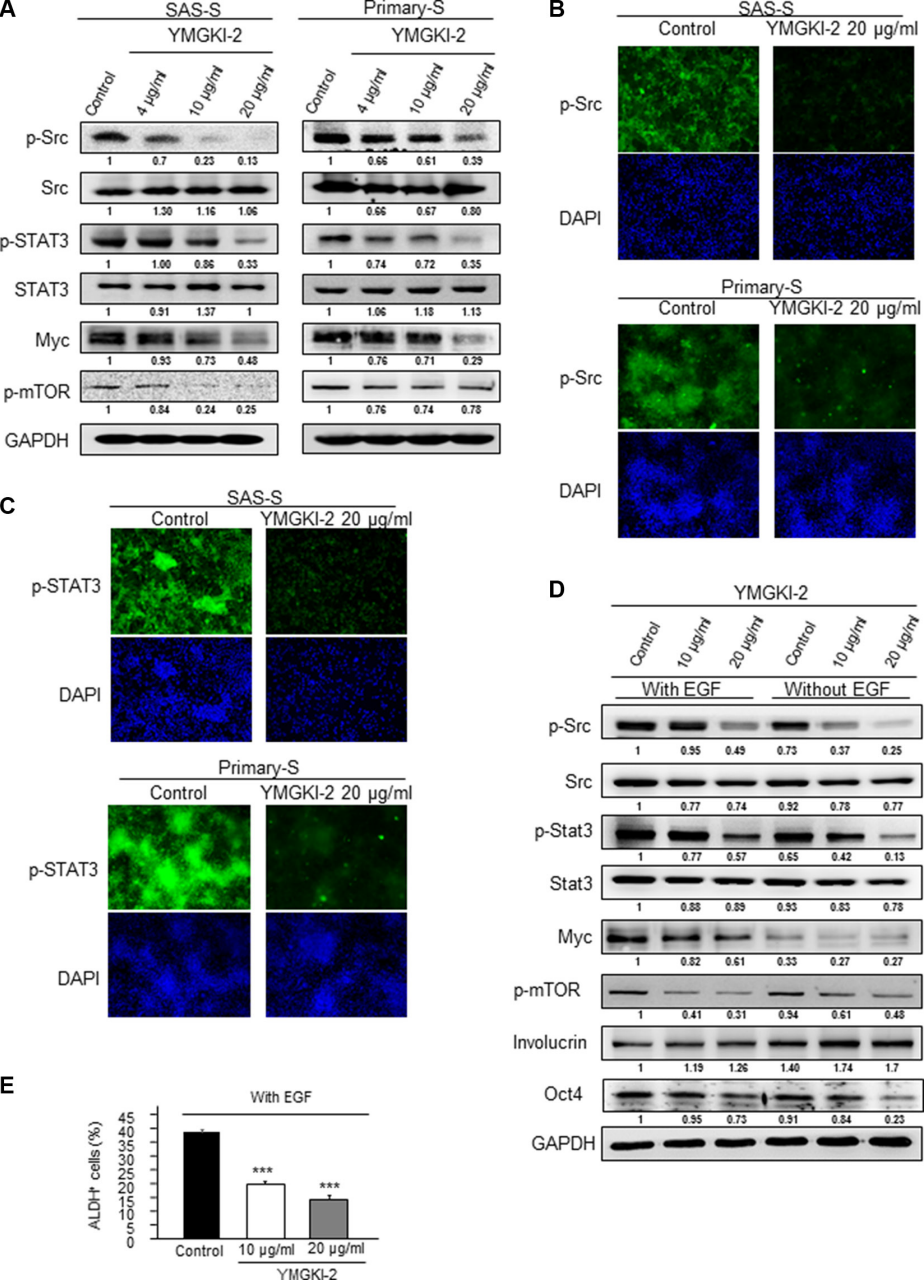
Inactivation of STAT3 and Src activity by YMGKI-2 treatment (**A**) Crude cell extract proteins of YMGKI-2 treated sphere cells (SAS-S, OECM1-S and Primary-S) were collected and analyzed by immunoblotting against anti-p-Src, anti-Src, anti-p-STAT3, anti-STAT3, Myc, p-mTOR or anti-GAPDH antibodies as indicated. The immunoactive signal of GAPDH protein of different crude cell extracts was referred as loading control. Immunofluorescent photograph of SAS-S and Primary-S stained with the primary antibody p-Src (**B**) or p-STAT3 (**C**) and secondary antibody conjugated with FITC fluorescence dye. Blue fluorescence indicates DAPI nuclear staining. (**D**) SAS cells treated with the YMGKI-2 (10 or 20 μM) or control cells (ethanol) in the absence or presence of 20 ng/ml EGF were cultivated under serum free medium, then, the crude cell lysates were collected and analyzed by immunoblotting with the indicated antibodies (The primary antibodies are listed in Supplementary Table S1). (**E**) SAS cells treated with the YMGKI-2 (10 or 20 μM) or control cells (ethanol) in the absence or presence of 20 ng/ml EGF were cultivated under serum free medium; afterwards; the intracellular ALDH activity was examined by ALDEFLUOR™ flow cytometry-based assay. The bar graph quantitating the ALDH-positive cells.

YMGKI-2 (Ergone) is a metabolite derived from ergosterol peroxide by a set of multiple pathways [35]. In addition, YMGKI-2 and ergosterol peroxide have the similar chemical structures [36]. A recent study finds ergosterol peroxide suppresses the STAT3 and Src activation [37], and it is found that both Src and STAT3 activations are involved in maintaining CICs properties of head and neck cancer [38–40]. To further investigate the mechanistic effect interfered by YMGKI-2 treatment to reduce the CICs properties, we examined the Src and STAT3 activity of YMGKI-2 treated sphere cells. Immunoblot analyses and immunofluorescence staining both showed that the level of phospho-Src and phospho-STAT3 protein was diminished in YMGKI-2 treated sphere cells (Figure 6A, 6B and 6C). Previous studies show that Src can activate STAT3/myc and mTOR pathway [41, 42], herein, we showed that YMGKI-2 treatment also reduced the expression of myc and p-mTOR (Figure 6A). To further verify the inhibitory effect of YMGKI-2 on these pathways in CICs, we cultured SAS cells in the presence of EGF (20 ng/ml) to induce the conversion of non-CSC to CSC via Src/STAT3 activation [43]. Accordingly, EGF stimulation increased the expression of phospho-Src, phospho-STAT3 and Myc (Figure 6D). However, YMGKI-2 treatment markedly decreased EGF-induced Src/Stat3 phosphorylation, and expression of stemness markers (Oct4 and ALDH activity) in sphere cells (Figure 6D and 6E). These data suggest that YMGKI-2 treatment impaired CICs properties through inactivating the Src and STAT3 pathways.

### Dual inhibition of STAT3 and Src activity as a new strategy for targeting CICs

**Figure 7:**
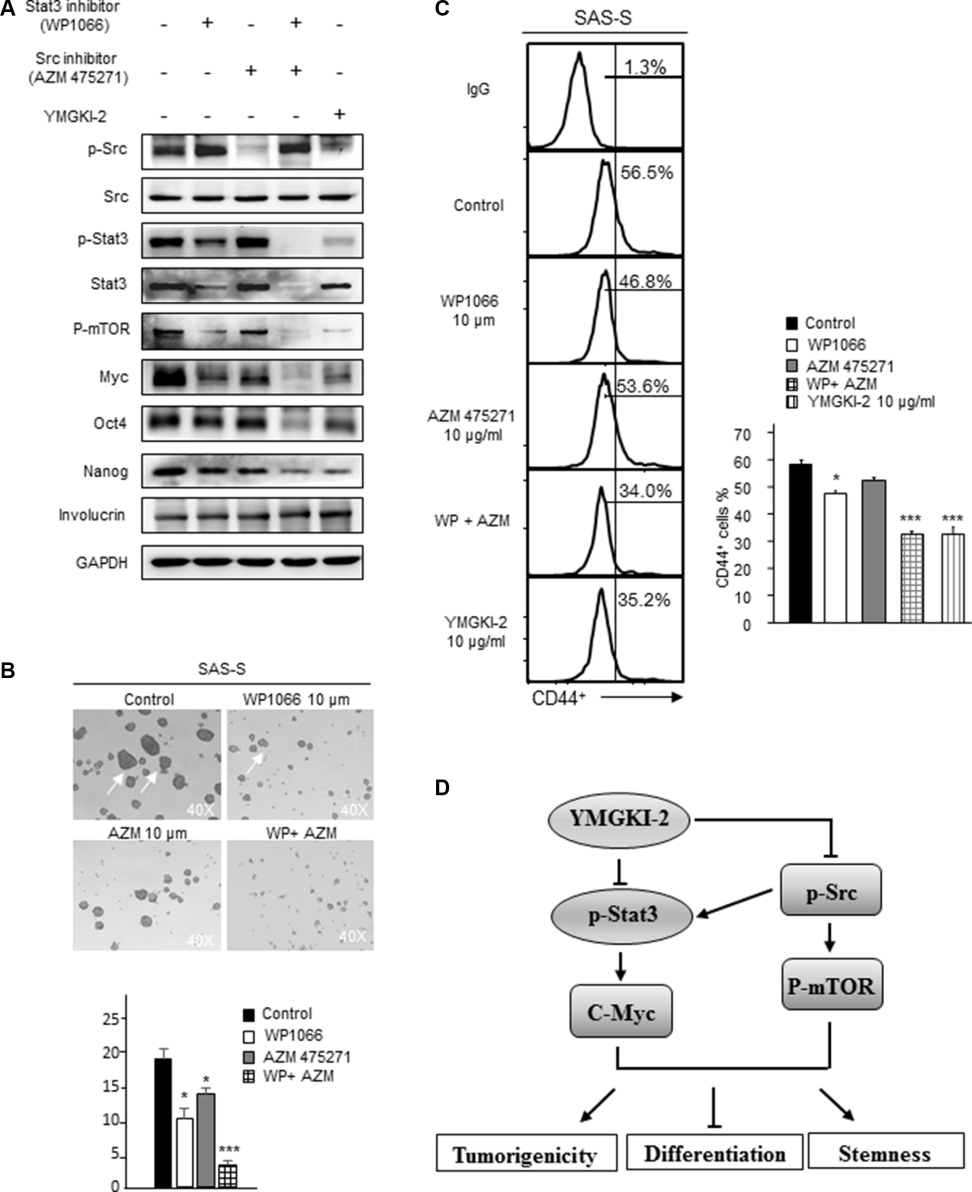
Combined treatment with Src and STAT3 inhibitors diminished the sphere formation ability and CIC marker expression of HN-CICs (**A**) SAS-Sphere cells were either singly treated or co-treated with Src (AZM 475271; 10 μM) and STAT3 inhibitors (WP1066; 10 μM) for 24 hr. Afterwards; the crude cell lysates were collected, and the protein level of pluripotent stemness markers (Oct4 and Nanog) and epithelial differentiation marker (Involucrin) was assessed by western blot. (**B**) Single cell suspension of sphere cells was either singly treated or co-treated with Src and STAT3 inhibitors for 24 hr, then, the sphere formation ability of treated sphere cells was assessed after 14 days. Arrows indicating the sphere cells. (**C**) CD44 positive cells in drugs treated cells were measured by FACS analyses. (**D**) Hypothetical model of the effects of YMGKI-2 on inhibition of stemness properties and tumorigenicity, but promoting differentiation of head and neck cancer initiating cells (HN-CICs). YMGKI-2 directly or indirectly inhibits the Src/Stat3 pathways which play the important roles in maintaining CICs properties of head and neck cancer.

In Figure 6, we showed that YMGKI-2 treatment reduced the self-renewal ability and enhanced the differentiation of CICs by dual inhibition of STAT3 and Src activity. Consequently, we wanted to address whether combined treatment with STAT3 and Src inhibitors (AZM 475271 and WP1066) would abrogate the stemness properties of HN-CICs. As shown in Figure 7A, the sphere cells under single treatment with STAT3 or Src inhibitors displayed decreased expression of stemness proteins (Oct-4 and Nanog). In addition, the sphere formation ability of HN-CICs under the single inhibition of STAT3 or Src activity was also significantly abolished (Figure 7B, *p* < 0.05). Nevertheless, the combined treatment of STAT3 or Src inhibitors showed the most significant effect to enhance the expression of differentiation marker (Involucrin) but reduced the expression of CIC marker (Oct4, Nanog and CD44) in sphere cells (Figure 7A and Figure 7C). Additionally, dual treatment of STAT3 or Src inhibitors also displayed the inhibitoriest effect on the sphere formation ability of HN-CICs (Figure 7B, *p* < 0.005). Next, we want to determine whether expression of STAT3 or Src can be used as the prognostic markers in HNSCC. As shown in Supplementary Figures S2A and S2B, we found that compared to the normal tissues the HNSCC patients' tumor tissues displayed higher expression of STAT3 or Src.

Overall, these data demonstrate that treatment of YMGKI-2 inhibits, at least, two signaling pathways (STAT3 and Src) to reduce the stemness properties and tumorigenicity but to enhance differentiation of HN-CICs (Figure 7D). Dual inhibition of STAT3 and Src activity may be a future alternative HNSCC treatment on targeting HN-CICs.

## DISCUSSION

In this present study, we demonstrated that YMGKI-2 treatment reduced the subpopulation of CICs such as ALDH^+^ and CD44^+^ cells in HNSCCs (Figure 1). Moreover, YMGKI-2 treatment reduced the self-renewal ability and down-regulated the expression of stemness genes in sphere cells with enriched HN-CICs (Figure 2C and 2D). We also found that YMGKI-2 treatment could promote cell differentiation and abrogate *in vitro* malignancy but not cause cytotoxicity of CICs (Figures 2A and 3). In nude mice xenograft model, YMGKI-2 pre-treated cells showed less tumor-initiating activity (Figure 4A). Additionally, YMGKI-2 post-administration also suppressed the HNSCC derived tumor growth in tumor-bearing mice (Figure 4B). Interestingly, YMGKI-2 effectively restored the synergistic chemosensitivity to conventional chemotherapeutic drugs in enriched HN-CICs (Figure 5). Finally, we observed that YMGKI-2 treatment inactivated the Src and STAT3 pathways (Figure 6). Activation of Src and STAT3 signaling pathway is involved in self-renewal ability and maintaining CICs properties in head and neck cancer [38, 39]. Our data suggests that YMGKI-2 treatment induces differentiation of CICs by inhibiting the dual Src and STAT3 signaling pathway.

CICs, a more resistant and malignant subpopulation of cancer cells, are considered a novel therapeutic target in cancer treatment. Elimination of CICs apparently requires exhaustion of stemness and promotion of differentiation by targeting self-renewal properties. Thus, it has been reported colorectal CICs/CSCs are induced differentiation and increased their response to chemotherapy by bone morphogenetic protein 4 (BMP-4) [44]. Moreover, such as resveratrol, abexinostat and curcumin were able to cause impairment of CIC properties, induce CIC differentiation and reduce tumor malignancy through inhibiting self-renewal signaling pathways [45–48]. In our previous study, we have demonstrated HN-CICs possess stemness properties, which is characterized by up-regulation expression of self-renewal gene Oct-4 and Nanog and differentiation ability [8]. Our present findings suggest that reduction of CIC subpopulation and decreased growth and tumorigenicity of HNSCC cell-derived tumor by YMGKI-2 treatment were mediated through the inhibitory self-renewal ability and induced differentiation (Figures 1, 2 and 4).

Mechanistically, we found that YMGKI-2 treatment inhibited the Src and STAT3 pathways in sphere cells (Figure 6). Further, dual treatment of STAT3 and Src inhibitors diminished the sphere formation ability and CIC marker expression more than the single inhibitor treatment in sphere cells (Figure 7). Therefore, dual inhibition of STAT3 and Src activity may be a future alternative HNSCC treatment on targeting HN-CICs.

STAT3 is an important component of maintaining self-renewing processes in several malignancy diseases [49], including head and neck cancer. Lee et al. show liver tumor-initiating cells drive self-renewal and tumorigenicity through STAT3-mediated Nanog up-regulation [50]. Chen et al. show that inhibition of STAT3 activity suppresses CIC properties and enhances therapeutic effect in HNSCC [51]. Src, a classical non-receptor tyrosine kinases is an important components of signal transduction pathways to control tumor growth, motility and therapeutic resistant [52]. Christoffer Tamm and colleagues have shown that inhibition of Src kinase activity promotes embryonic stem cell to differentiated state, including the decreased expression of Oct3/4 and Nanog [53]. In our previous study, we have demonstrated CD133/Src axis regulated CIC properties and epithelial-mesenchymal transition of head and neck cancer [38]. Our present findings suggest that YMGKI-2 treatment decreases CIC properties and malignancy through dual inhibition of STAT3 and Src activity in HN-CICs. Overall, future research delineates the detail mechanism of how YMGKI-2 inhibits kinase activity, and how downstream target influences the stemness properties of CIC remain to be determined.

We and others have demonstrated that CICs are more resistant to conventional chemotherapeutic agents such as cisplatin and 5-FU (Figure 5) [45]. Single treatment with YMGKI-2 promoted cell differentiation but not induced cell death in HN-CICs or HNSCCs (Figure 1E). It is reasonable that YMGKI-2 could enhance the chemosensitivity of CICs through induced differentiation. Herein, we confirmed that combinatorial treatment of YMGKI-2 and chemotherapeutic agents (cisplatin and 5-FU) exhibited the enhanced cytotoxicity in HN-CICs (Figure 5). Further, YMGKI-2 treatment also restored the drug sensitivity to cisplatin in cisplatin-resistant HNSCCs (Figure 5D). Our data suggest that co-treatment with YMGKI-2 along with a chemotherapeutic agents may improve the future treatment of head and neck cancer.

The anti-tumor effect of YMGKI-2 on HNSCC was evaluated in immunocompromised mice (Figure 4B). Other also demonstrate that YMGKI-2 possesses pharmacological activities *in vivo* and *in vitro* [54–57]. Together, these studies suggest that YMGKI-2 is a potential drug for disease treatment. However, the poor water solubility of YMGKI-2 makes it difficult to dissolve in the hydrous solution and lessens its bioavailability [56]. Therefore, it is necessary to develop an efficient pharmacological delivery methodology of YMGKI-2 to enhance its future clinical application.

In summary, our present research shows YMGKI-2 could reduce the subpopulation of CIC and suppress cancer malignancy through induced differentiation in HNSCCs. Further, clinical therapies could be developed from the combined treatment with YMGKI-2 to improve the head and neck patients' outcome in the future.

## MATERIALS AND METHODS

### Extraction, isolation, purification, and structure determination of single compounds from the *Antrodia cinnamomea* mycelias (ACMs)

ACMs were obtained from the Biotechnology Center, Grape King Inc., Taoyuan County, Taiwan [58]. The *Antrodia cinnamomea* mycelia extract (ACME) was extracted with 95% EtOH from ACMs. YMGKI-2 (Ergosta- 4,6,8(14),22-tetraen-3 one; also named Ergone) from ACME and the chemical structure of the purified chemicals were performed and determined by Dr. Chien-Chih Chen (Hungkuang University, Taichung, Taiwan) [26]. The structure elucidation of YMGKI-2 by ^1^H NMR and ^13^C NMR was provided in Supplementary Figure S3A and S3B. Ethanol (EtOH) was used as a drug solvent.

### Cell lines

SAS tongue carcinoma cells, human HNSCC cell lines, obtained from the Japanese Collection of Research Bioresources (Tokyo, Japan) were cultured in DMEM medium containing 10% fetal bovine serum (Grand Island, NY) [59]. Human gingival squamous carcinoma cells (OECM1) were provided from Dr. C. L. Meng (National Defense Medical College, Taipei, Taiwan) and grown in RPMI medium containing 10% fetal bovine serum. Cells were cultured at 37°C containing 5% CO2. Short tandem repeat (STR) genotyping had been performed for authentication of used cell lines by Genelabs Life Science Corporation (Taipei, Taiwan).

### Enrichment of CICs from HNSCCs

The two HNSCC cell lines SAS and OECM1 were seeded at a density of 7.5 × 10^4^ live cells/10-mm dish, then cultured in selection medium consisting of serum-free DMEM/F12 medium (GIBCO), N2 supplement (GIBCO), 10 ng/mL human recombinant basic fibroblast growth factor-basic (bFGF) and 10 ng/mL Epidermal Growth Factor (EGF) (R&D Systems, Minneapolis, MN). The medium was changed every other day until the tumor sphere formation was achieved to enrich the CICs from SAS or OECM1 in about 4 weeks [8]. We named the enriched CICs from SAS and OECM1 as SAS-S or OECM1-S, respectively.

### ALDH activity assay

The ALDEFLUOR kit (Stem Cell Technologies, Durham, NC, USA) was used to examine the ALDH enzymatic activity. The ALDH activity was determined according to the protocol described in ALDEFLUOR Kit, and the intensity of intracellular fluorescence was measured by FACS Calibur apparatus (Becton Dickinson, San Diego, CA).

### Establishment of the primary CICs (primary-S) from HNSCC patient

This research follows the tenets of the Declaration of Helsinki and all samples were obtained after informed consent from the patients. All of used clinical samples were approved and in accordance with the institutional review board (IRB), Taipei Veterans General Hospital. Primary CICs were established from HNSCC patient tumor that derived from surgical specimens. The primary CICs (primary-S) were cultured and formed sphere in serum-free DMEM/F12 medium (GIBCO), N2 supplement (GIBCO), 10 ng/mL Epidermal Growth Factor (EGF) and 10 ng/mL human recombinant basic fibroblast growth factor-basic (bFGF) (R&D Systems, Minneapolis, MN).

### Establishment of cisplatin-resistant cell line

To generate cisplatin-resistant cells from HNSCC SAS cells, the parental SAS cells were treated to a low dose of 5 μM cisplatin (Sigma Aldrich, St Louis, MO, USA) for 48 h, and then the cells were allowed to recover over 20 days with fresh medium and repeat two times. Those cells were then subsequently subjected to 10 μM cisplatin for 72 hr and recover until sphere cells were seen. The CICs properties of cisplatin resistant (SAS-CisPt^R^) cells have been elucidated [9].

### Cell viability and chemo-resistance assay

Cells were seeded into 24-well culture plates at 1 × 10^4^ cells/well for 24 hours. Then the test drugs were added to the culture medium for 72 hr. Subsequently, 5 μl of MTT solution (4 mg MTT/ml PBS) was added to each well and the cells were further incubated at 37°C for 3 hours until a purple formazan was visible. The staining solution was removed and 200 μl DMSO was added at room temperature in the dark for 30 min. The absorbance of DMSO solution was detected with a microtiter plate reader at 560 nm. The cell viability ratio was calculated as OD560 of experimental groups/OD560 of control groups.

### Anchorage independent growth assay

Each well (35 mm) of a six-well culture dish was coated with 2 ml bottom agar (Sigma-Aldrich) mixture (DMEM, 10% (v/v) FBS, 0.6% (w/v) agar). After the bottom layer was solidified, sorted cells were cultured in 2 ml top agar-medium mixture (DMEM, 10% (v/v) FBS, 0.3% (w/v) agar), and the dishes were incubated at 37°C for 2 weeks. Subsequently, plates were stained with 0.005% Crystal Violet, then the stained colonies were counted. The number of total colonies was counted over five fields per well for a total of 15 fields in triplicate experiments.

### *In vivo* tumorigenic assay

All animal studies were approved and in accordance with the Institutional Animal Care and Use Committee (IACUC) of National Yang-Ming University, Taipei, Taiwan (IACUC approval No. 1001223 and No. 991235). SAS cells were subcutaneously injected into the subcutaneous of nude mice (6–8 weeks). Tumor volume (TV) was calculated using the following formula: (Length × Width 2)/2.

### *In vitro* migration assay

Cells treated or untreated with YMGKI-2 for 24 hr were subject to migration assay. For migration assays, 2 × 10^5^ cells were added onto the top chamber of a Transwell (Corning, Acton, 8.0 μm pore size) in medium without serum. In the lower chamber, medium containing higher serum (10% FBS) was applied as a chemoattractant. After 24 h of incubation, cells were fixed with 3.7% formaldehyde and the remaining cells on the upper side of the Transwell were removed with a cotton swab. Cells on the lower surface of the membrane were stained with crystal violet staining solution and Hoechst 33258 (SigmaAldrich) to show the nuclei. The migratory capacity was determined by counting fluorescent cells using a fluorescence microscope (Carl Zeiss, Oberkochen, Germany). The number of fluorescent cells were counted in a total of five randomly selected fields.

### Drug synergism analysis

Chou-Talalay method [60] was used to determine the synergistic effect of drug combination as described by Trudel et al [61]. The follow equation used to calculate the synergistic effect: Combination index (CI) = (D)A/(Dx)A + (D)B/(Dx)B; (D)A and (D)B: dose of drug A and B cause the x effect in combined treatment; (Dx)A and (Dx)B: dose of drug A and B cause the same x effect in alone treatment. (CI = 0: additive effect, CI < 1.0: synergistic effect, CI > 0: antagonistic effect) [61].

### Statistics

The unpaired Student *t-test* was used for statistical analyses. A *p-value* less than 0.05 was considered as statistical significance.

## SUPPLEMENTARY MATERIALS FIGURES AND TABLE


