# Supplementary material for: Targeting cancer initiating cells by promoting cell differentiation and restoring chemosensitivity via dual inactivation of STAT3 and Src activity using an active component of Antrodia cinnamomea mycelia
Source: Oncotarget. 2016 Sep 22;7(45):73016–31. doi: 10.18632/oncotarget.12194 (PMC5341960; doi:10.18632/oncotarget.12194)
Supplement: Supplementary file 1 [file oncotarget-07-73016-s001.pdf]

Targeting cancer initiating cells by promoting cell differentiation and restoring chemosensitivity via dual inactivation of STAT3 and src activity using an active component of *antrodia cinnamomea* mycelia

Supplementary Materials

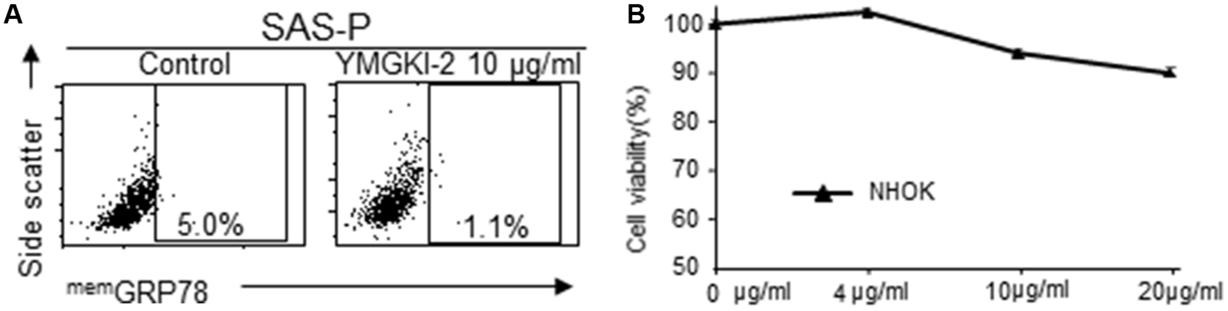

Supplementary Figure S1: Efficient elimination of memGrp78+ HNSCC cells by YMGKI-2 treatment. (**A**) memGrp78+ of YMGKI-2 or EtOH control treated HNSCC cells were analyzed by flow cytometry. (**B**) The cell viability of YMGKI-2-treated normal human oral keratinocytes (NHOKs) was measured by MTT assay.

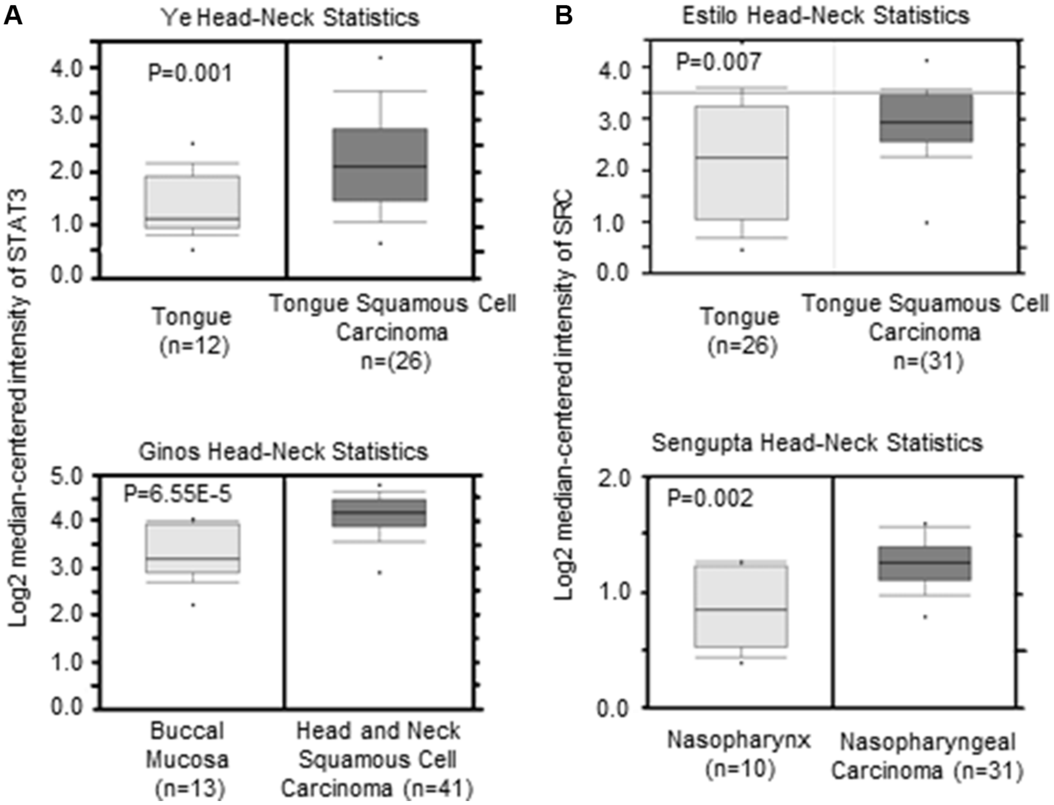

Supplementary Figure S2: Clinicopathological analysis of HNSCC according to the expression of Stat3 and Src. Expression levels of Stat3 (**A**) and Src (**B**) in normal and HNSCC from patients, respectively. These datasets were obtained from the Oncomine database.

A ^{1}H-NMR

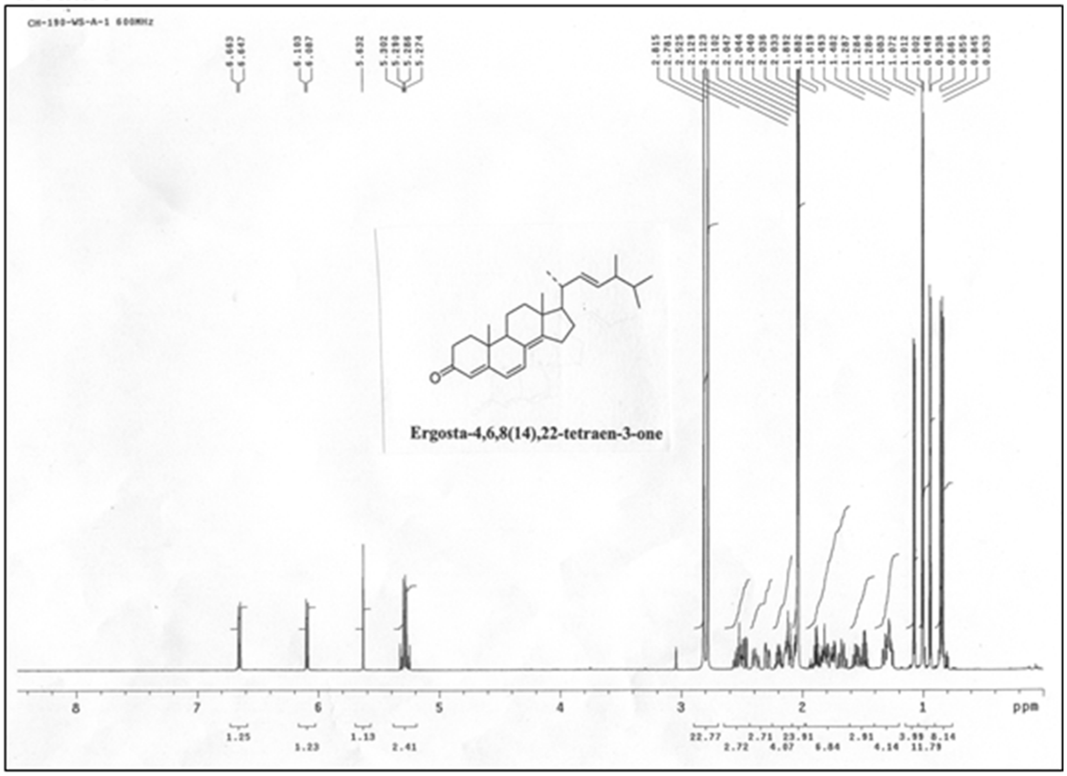

B ^{13}C-NMR

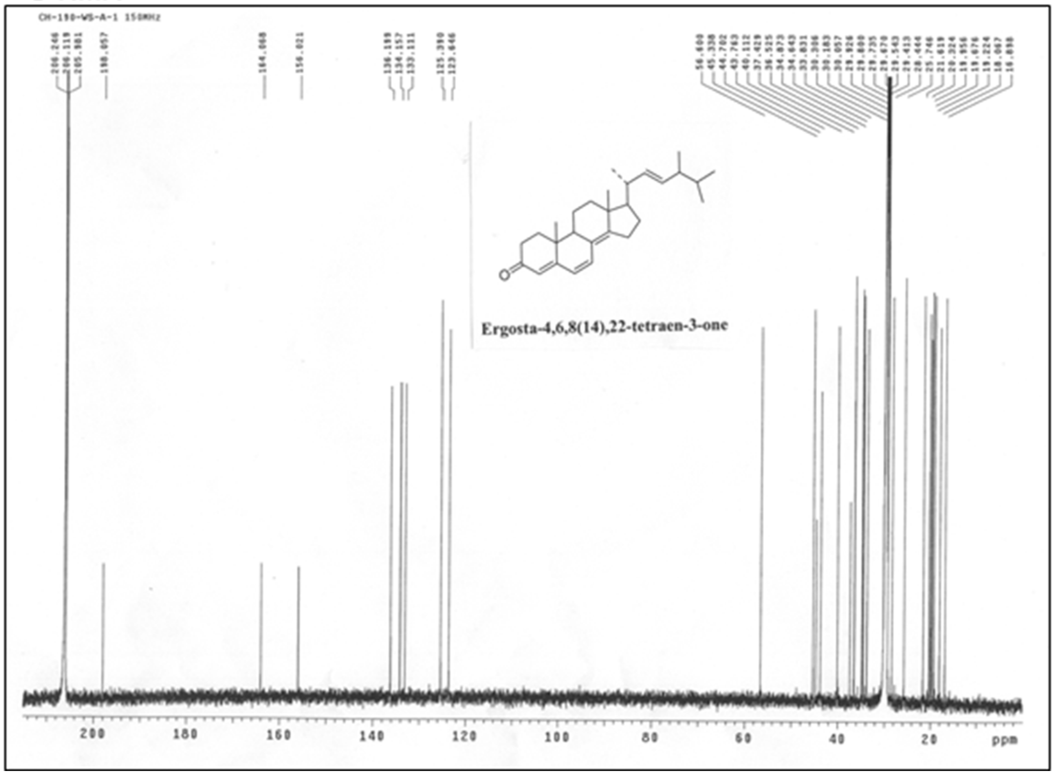

Supplementary Figure S3: NMR data for YMGKI-2 (Ergosta-4,6,8(14),22-tetraen-3-one). (**A**) ^{1}H-NMR spectrum of YMGKI-2. (**B**) ^{13}C-NMR spectrum of YMGKI-2.

Supplementary Table S1: Antibodies

Antibodies and reagents	Product company	Catalog No.	Dilution
**Antibodies**			
CD44	R&D	FAB5088A	1:25
Grp78	BD Transduction Laboratories™	610979	1:100
Nanog	Millipore	AB9220	1:1000
Oct-3/4 (H-134)	Santa cruz	sc-9081	1:1000
GAPDH	Millipore	AB2302	1:20000
Involucrin	Sigma	I 9018	1:1000
CK-18	Millipore	MAB3234	1:2500
p-Stat3	Cell Signaling	9145	1:1000
Stat3	Cell Signaling	12640	1:1000
p-Src	Cell Signaling	2101	1:1000
Src	Millipore	05-184	1:1000
c-Myc	Santa cruz	sc-764	1:1000
p-mTOR	Cell Signaling	2971	1:1000
